# Serotonin 5-HT_2A _and 5-HT_6 _receptors in the prefrontal cortex of Alzheimer and normal aging patients

**DOI:** 10.1186/1471-2202-7-36

**Published:** 2006-04-27

**Authors:** Dietrich E Lorke, Gang Lu, Eric Cho, David T Yew

**Affiliations:** 1 Department of Anatomy, Faculty of Medicine and Health Sciences, UAE University, Box 17666, Al Ain, United Arab Emirates; 2 Department of Anatomy, Chinese University of Hong Kong, Shatin, N.T., Hong Kong; 3 Kunming Medical College, Kunming, Yunnan, China

## Abstract

**Background:**

It has been hypothesized that alterations of the serotonergic system contribute to neuropsychiatric symptoms in Alzheimer disease (AD). Cellular expressions of the two serotonergic receptors 5-HT_2A _and 5-HT_6 _have therefore been determined by immunohistochemistry in the prefrontal cortex of patients with AD (n=6) and normal age-matched controls (n = 7).

**Results:**

In normal aging patients, 5-HT_2A _label was mainly observed in large pyramidal cells, but to a lesser extent also in small pyramidal cells and in stellate cells of cortical layers II-VI. In AD, a similar distribution was observed, but density of positive cells was significantly reduced by 33%. In aging control patients, the 5-HT_6 _receptor was expressed by pyramidal cells and occasional stellate cells, not only of layers II-V, but also of layer I, where a distinct label was observed in neurons and surrounding fibers. 5-HT_6 _receptor expression in AD patients had the same pattern, but was significantly decreased by 40%.

**Conclusion:**

Our results indicate that a decline in neurons expressing 5-HT_2A_, but also 5-HT_6 _receptors may play a role in the etiopathology of neuropsychiatric symptoms in AD.

## Background

Alzheimer disease (AD), the most common cause of dementia in the elderly, is clinically characterized by progressive cognitive impairment associated with severe neuropsychiatric disturbances. These behavioral and psychological symptoms of dementia (BPSD) include hallucinations, delusions, aggressive behavior, overactivity, anxieties and affective disturbances [1,2]. Whereas the decline in cognitive functions can be largely related to cholinergic dysfunction arising from disruption of basal forebrain cholinergic pathways (cholinergic hypothesis) [1,3,4], impaired balance between several neurotransmitters has been implicated in the pathogenesis of BPSP [2,5-7], with serotonin (5-HT) playing a pivotal role [2,8-10]. The actions of 5-HT are mediated through seven major receptors classes, 5-HT_1–7_, comprising a total of 14 distinct mammalian 5-HT receptor subtypes (for review, see [11]). The 5-HT_2A _receptor has attracted most interest because of its possible participation in behavioral alterations in AD. 5-HT_2A _is localized in the cortex and caudate and is involved in anxiety [10]. 5-HT_2A _receptors mediate the psychotomimetic effects of hallucinogens [11-13], and alterations in binding characteristics to this receptor have been observed in the prefrontal cortex of patients suffering from psychiatric diseases, e.g. schizophrenia [14,15], depression [16] and suicide [17]. Electrophysiological evidence suggests that 5-HT_2A _receptors are involved in the 5-HT-induced increase in excitatory postsynaptic potentials [12,18] and play an important role in the working memory process [13]. There is indication that 5-HT_2A _also participates in the etiopathology of AD. Serotonin increases the secretion of amyloid precursor protein (APP) through activation of 5-HT_2A _receptors [19]. 5-HT_2A _receptor binding is decreased in AD (for review, see [9,10]), and polymorphic variations have been described for the *5-HT*_2*A *_gene that may be risk factors for hallucinations [20], aggression [21] and major depression [22] in AD. Much less is known about the 5-HT_6 _receptor, the most recent 5-HT receptor to be identified. Many antidepressants and antipsychotics are antagonists of the 5-HT_6 _receptor [15,23]. It has been shown to influence acetylcholine release in the frontal cortex [24] and may play a role in cognition deficits and in some form of anxiety [23]. A recent autoradiographic study examining [^125^I]-SB-258585 binding in autopsy specimens of AD patients indicates lowered 5-HT_6 _receptor density in the frontal and temporal cortices [5]. Association of a *5-HT*_6 _receptor gene polymorphic variant and late-onset AD has been observed in the Chinese population [25], but not in Germans [26].

Surprisingly, although 5-HT_2A _and 5-HT6 receptor binding in AD has been studied extensively [2,5,9,10], there is no information on the expression of these receptors at the cellular level. We have therefore examined the expression of the 5-HT_2A _and 5-HT_6 _receptors in the brains of AD and of normal aging control patients by immunohistochemistry. Because the prefrontal cortex is of particular importance for the etiopathology of BPSD [1,8,12,14,16,27-31] and because neuropsychiatric symptoms in AD are associated with reduced metabolism [32,33] and perfusion [34] in Brodmann area 10, we have chosen this brain region for examination.

## Methods

### Tissue samples

The brains were obtained from a total of 13 autopsy cases with the consent of a close relative and with full approval by the ethical committees of Guangzhou hospital authorities and of the Chinese University of Hong Kong. Six of the specimens were from individuals with clinically and pathologically diagnosed senile dementia of the Alzheimer's type (mean age: 88 years, see table 1), seven specimens were from individuals, matched for age, gender and *postmortem *delay, who had no history of neurological diseases (normal control). Drug history was recorded for all patients, who had only received antibiotic treatment, but no psychopharmacological medication. Alzheimer Disease was clinically diagnosed according to the "National Institute of Neurological and Communicative Disorders and Stroke and the Alzheimer's Disease and Related Disorders Association" (NINCDS-ADRA) criteria and the "Diagnostic and Statistical Manual of Mental Disorders", Fourth Edition, (DSM-IV-R) criteria.

Whole brains were removed with an average *postmortem *delay of six hours, and tissue samples of the anterior prefrontal cortex (Brodmann's area 10) were dissected. Specimens were fixed overnight in 4% paraformaldehyde in phosphate-buffered saline (PBS; ph 7.4), dehydrated in graded ethanol, cleared with xylene and embedded in paraffin. 6-μm-thick serial sections were cut in the coronal plane, 90° perpendicular to the surface to avoid sectioning artifacts. Two sets of slides were obtained for each individual.

### Immunohistochemistry

All steps were carried out at room temperature unless stated otherwise. Mounted sections were dewaxed, rehydrated and predigested with 0.1% trypsin (BDH Laboratory Supplies, Poole, U.K.) in 0.05M tris buffered saline containing 0.1% CaCl_2 _(pH 7.6; 37°C; 20 min) followed by two rinses (5 min) in 0.01M PBS for antigen retrieval. The endogenous peroxidase activity was blocked with 0.3% hydrogen peroxide in absolute methanol for 30 min, followed by another three rinses in PBS (5 min each). To suppress non-specific binding, sections were then incubated for 1h in 2 % normal blocking serum (Vectastain^R ^ABC Kit, Vector Laboratories, Burlingame, CA) in 0.3 % triton/PBS. Thereafter, sections were incubated overnight with the primary polyclonal antibodies: either goat anti-human serotonin 2A receptor (Santa Cruz sc-15073, Santa Cruz Biotechnology Inc., Sta. Cruz, CA) or goat anti-human serotonin 6 receptor (Santa Cruz sc-26668). The sections were then washed with three rinses of PBS containing 0.05 % Tween 20 (5 min each) and incubated with biotinylated secondary antibody in blocking solution (1:200) for 30 min (PK6105, anti-goat IgG, Vectastain^R ^ABC Kit, Vector Laboratories, Burlingame, CA). Sections were washed three times in PBS again (5 min) and incubated with ABC Reagent (Vectastain^R ^ABC Kit, Vector Laboratories, Burlingame, CA) for 30 min. The immunocytochemical staining signals were visualized by incubating the sections in 0.05% of the substrate 3'3'-diaminobenzidine tetrahydrochloride (DAB) in PBS containing 0.01% H_2_O_2_. All stainings included negative controls with omission of the primary antibody, which did not show any immunoreaction. The sections were washed with distilled water and then counterstained with 0.5% cresyl fast violet in 0.1M sodium acetate (pH 3.5, 5 min). They were differentiated with 95% ethanol, dehydrated, cleared and covered in Permount^® ^(Fisher Scientific, Hampton, VA). Additional sections were stained with routine methods, including H&E, Nissl and Bielschowsky silver impregnation for tangle staging [35,36].

All cases underwent a standardized post-mortem neuropathological assessment of AD using established criteria including Braak staging [35]. Four brain regions were examined: prefrontal (area 10), occipital (area 17), entorhinal (area 28) and hippocampal cortices. Diagnosis was confirmed by the characteristic presence of amyloid plaques, neurofibrillary tangles (NFT) and neuronal degeneration. In addition, the presence of β-amyloid in the plaques was ascertained by immunohistochemistry using the rabbit polyclonal Anti-Amyloid Peptide β, Cleavage site 42 (Aβ42), antibody (A1976, Sigma, St. Louis, MU) [37]. All AD cases had neocortical NFT with involvement of both isocortical association areas and additional involvement of primary cortical (area 17) areas (Braak stage V-VI). In control patients, deposition of NFT was negligible in the entorhinal cortex and absent in the hippocampus as well as the neocortex (Braak stage = I). To exclude Dementia with Lewy bodies, all brain specimens were scrutinized for Lewy bodies, and only cases devoid of Lewy bodies were included in the study.

### Statistics

For qualitative and quantitative evaluation, sections were observed under a photomicroscope (Axioplan 2 Photomicroscope, Zeiss, Germany). The number of 5-HT_2A_- and 5-HT_6_-immunoreactive cells was counted in 30 random selected 700 μm^2 ^fields of normal aged and AD patients. 4–5 sections per individual were evaluated, sparing areas containing amyloid plaques. It has been shown in AD patients [38] that astrocytes in and around Aβ plaques are strongly positive for 5-HT_2A _receptor protein, whereas astrocytes in control patients do not display any 5-HT_2A _immunoreaction. Amyloid plaques were therefore excluded from the quantitative assessment, because the aim of our study was to count neurons expressing 5-HT receptors. Measurements were expressed as means ± SEM. For statistical comparison, the values were subjected to a one-way analysis of variance (ANOVA), demonstrating that the four data groups exhibited equal variance, but were not distributed normally. We have then applied the One Way non-parametric ANOVA (Kruskal-Wallis test) and the Mann Whitney Rank sum test, yielding the same results. A p value below 0.05 was considered significant.

## Results

**Figure 1:**
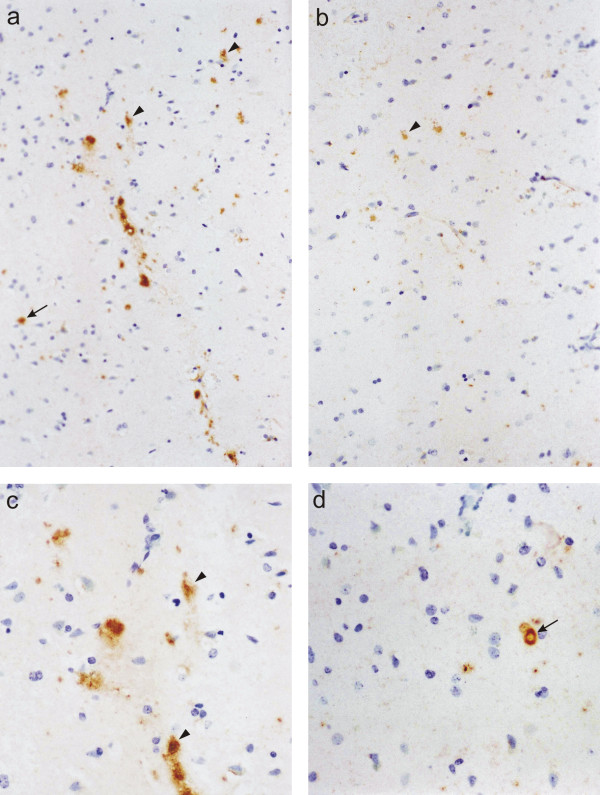
**5-HT_2A _receptor**. Immunoreaction for the 5-HT_2A _receptor in the prefrontal cortex of a normal aging (a, c) and an Alzheimer patient (b, d), cresyl violet counterstain. Both large pyramidal cells (arrowheads) and small interneurons (arrows) are stained. Note the reduction in labeled cells in the cortex of the Alzheimer patient (b, d). a, b: x200; c, d; x400.

**Figure 2:**
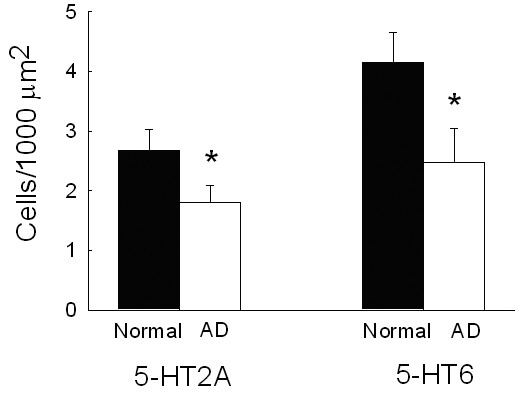
**Cell densities. **Numerical density of 5-HT_2A _and 5-HT_6 _receptor immunoreactive neurons in the prefrontal cortex of normal aging (n=7) and Alzheimer (AD) patients (n=6). Depicted are means ± SEM. Significant differences (p ≤ 0.05) are marked by an asterisk (*)

In the prefrontal cortex of normal aging patients, 5-HT_2A _receptor immunoreaction was observed in cortical layers II-VI. Large pyramidal neurons of layer V constituted the majority of immunoreactive cells, characterized by a strongly labeled cytoplasm and a moderately stained proximal part of the apical dendrite (Figs. 1a, c). In addition, smaller pyramidal cells and scattered stellate-shaped cells in the other layers were immunoreactive. Stellate cells had a strongly stained cytoplasm and labeled thin processes radiating to the side of the ovoid cell bodies and most likely represented interneurons. A few multipolar cells in layer VI were also 5-HT_2A _receptor positive. In the underlying white matter, occasional 5-HT_2A _receptor immunoreactive fiber bundles were observed. AD patients showed a similar distribution of 5-HT_2A _receptor label in the prefrontal cortex, i.e. staining in the cell bodies and apical dendrites of large pyramidal cells and in the soma and tender processes of a few scattered interneurons (Figs. 1b, d). However, numerical density of 5-HT_2A _receptor immunoreactive cells was significantly (p=0.001) decreased by 33% in the frontal cortex of AD patients (Fig. 2), as compared to normal aging patients. Reduction affected both pyramidal cells and cells of stellate morphology. In addition, labeled cells were also seen within and close to amyloid plaques. An association between 5-HT_2A _receptors and NFT was not observed.

**Figure 3:**
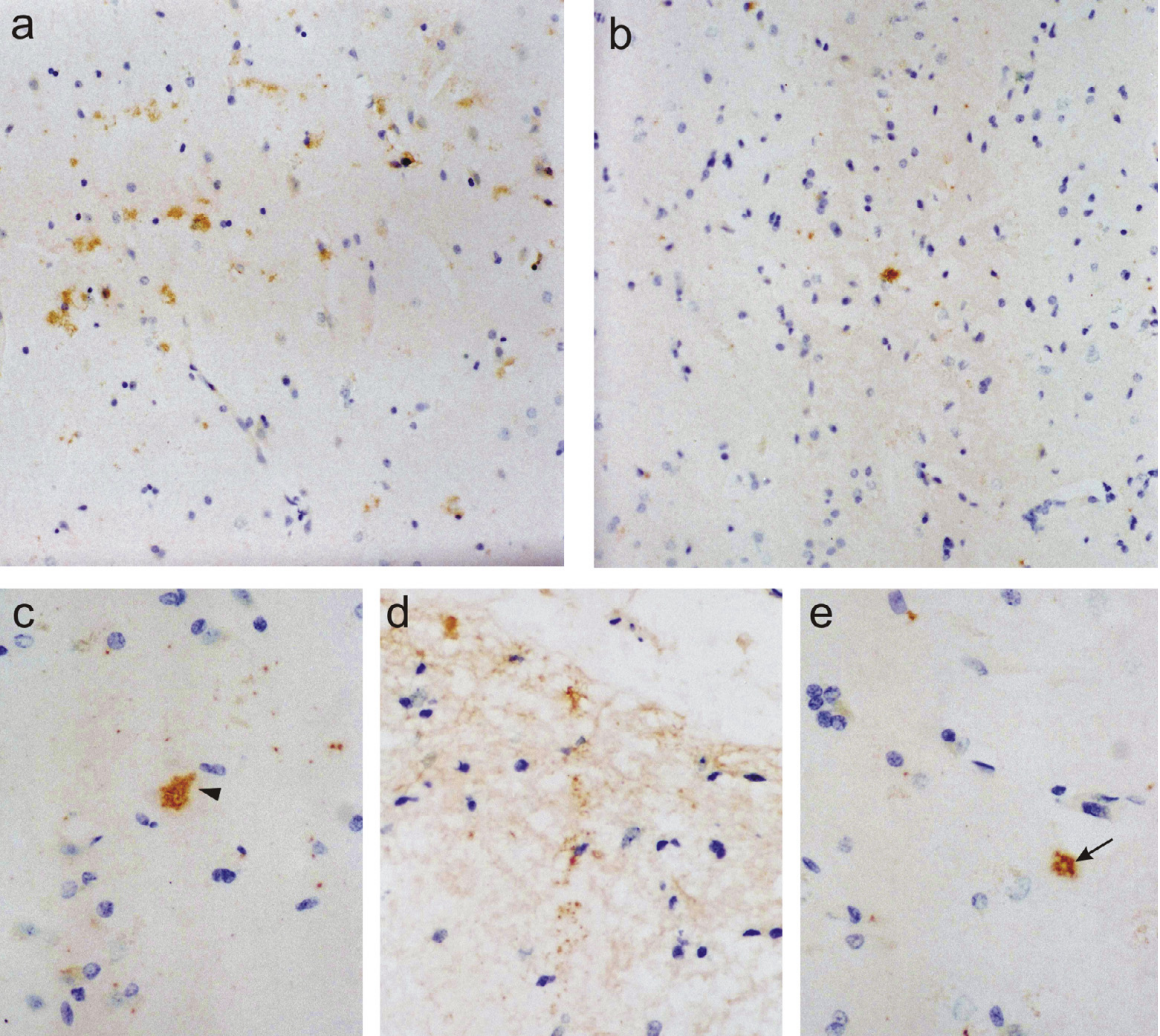
**5-HT_6 _receptor. **5-HT_6 _receptor immunoreactive cells in the prefrontal cortex of normal aging (a, c, d) and Alzheimer patients (b, e), cresyl violet counterstain. Label is observed in large pyramidal cells (arrowhead) and small interneurons (arrow). Note positive cells and dense immunoreactive fibers in the molecular layer (d). In the cortex of the Alzheimer patient (b, e), significantly fewer cells are labeled than in that of the normal aging patient (a, c, d). a, b: x200; c, d, e; x400.

Distribution of 5-HT_6 _receptor immunoreaction in the prefrontal cortex of normal aging patients was slightly different from that of the 5-HT_2A _receptor. As for the 5-HT_2A _receptor, the most prominent label was observed in the cell bodies and the proximal apical dendrites of large pyramidal neurons, and occasionally, the soma and fine processes of scattered stellate cells were stained as well (Figs. 3a, c). In addition, 5-HT_6 _receptor immunoreaction was also detected in neurons of the molecular layer (layer I). These cells had a bipolar strongly labeled cell body and numerous immunoreactive processes radiating into the molecular layer (Fig. 3d). In contrast, hardly any 5-HT_6 _receptor immunoreactive cells were observed in layer VI. Occasional immunoreactive fiber bundles were seen in the underlying white matter. In summary, density of 5-HT_6 _receptor positive neurons was significantly higher than that of 5-HT_2A _receptor immunoreactive cells (p=0.05). In the prefrontal cortex of AD patients, 5-HT_6 _receptor label showed the same overall distribution (Figs. 3b, e), but numerical density of 5-HT_6 _receptor immunoreactive cells was again significantly (p=0.001) reduced, as compared to normal aging patients (Fig. 2). Both pyramidal and stellate cells were decreased by about 40%. There was no association between 5-HT_6 _receptors and NFT.

## Discussion

The present study has been undertaken in order to determine changes in cellular distribution of two serotonin receptors, 5-HT_2A _and 5-HT_6_, in the prefrontal cortex of AD patients as compared to normal age-matched individuals. Our observation in aging (control) patients, showing 5-HT_2A _immunoreactivity in the cell bodies and the apical dendrites of pyramidal cells as well as in stellate-shaped cells, is consistent with previous studies in young humans [17], primates [18,39] and rodents [40,41]. However, we have detected relatively few labeled cells, and we are currently testing if the paucity in 5-HT_2A _receptors is attributable to the very advanced age of our patients or to the technique employed. A considerable age-related reduction in the number of cortical 5-HT_2A _binding sites in the frontal lobe has been described by numerous authors (see [9] for review). In contrast to 5-HT_2A_, relatively little is known on the distribution of the 5-HT_6 _receptor in the mammalian neocortex. 5-HT_6 _receptor binding sites have been detected by receptor autoradiography in the gray matter of the prefrontal cortex of healthy human adults [5,42], but there are no studies on the cellular level. We have been able to show that the 5-HT_6 _receptor is expressed both by pyramidal cells and by stellate-shaped cells located in cortical layers I-V. We have observed very little 5-HT_6 _immunoreaction in layer VI, but a distinct 5-HT_6 _label in layer I, with staining detected not only in a dense network of fibers, but also in neurons. This is somewhat at variance with a study on the rat neocortex, where 5-HT_6 _receptor mRNA has been detected by in-situ hybridization in layers II-VI, but not in layer I [43]. This discrepancy is, however, not surprising, given the marked inter-species differences described [44].

To our knowledge, this is the first immunohistochemical study of 5-HT_2A _receptor expression in AD. The reduction observed corroborates the results of previous 5-HT_2A _receptor binding studies showing decreased binding of ^3 ^[H] ketanserin [45-47] and ^3 ^[H] spiperone [48] in *postmortem *specimens of the frontal cortex of AD patients and in PET imaging studies [49]. Contrasting reports describing unaltered ^3 ^[H] ketanserin binding [50], which are at variance with our results, may be attributable to recent psychotropic medication, which had not been administered to our patients. Reduced 5-HT_2A _receptor binding in AD has been explained by a loss of interneurons [51]. In contrast, our immunohistochemical data indicate that both 5-HT_2A _immunoreactive pyramidal and stellate-shaped cells, i.e. interneurons, are affected in AD. Moreover, we have demonstrated that the density of neurons expressing the 5-HT_6 _receptor is reduced to a similar extent, corroborating autoradiographic studies revealing decreased binding to 5-HT_6 _receptors in the prefrontal cortex of AD patients [5]. Our observation of reduced density of neurons expressing 5-HT_2A _and 5-HT_6 _receptors, which is not accompanied by similar alterations in GABAergic markers [6], points at a selective vulnerability of neurons receiving serotonergic input. This decrease is associated with several other abnormalities of the serotonergic system in AD. A marked depletion in 5-HT and its metabolite 5-Hydroxyindole Acetic Acid (5-HIAA) has been described in the frontal and temporal cortices of AD patients [2,28,52], which is most likely attributable to a reduction in serotonergic projection fibers. The underlying cause appears to be a significant loss of neurons in the dorsal and median raphe nuclei [53,54], which are the source of serotonergic nerve terminals and which, in addition, are a preferential site for neurofibrillary tangle formation [55]. It is very likely that the decrease in neurons expressing 5-HT_2A _and 5-HT_6 _receptors is related to this loss of serotonergic fibers. However, it may either reflect a process of primary cortical degeneration [4,56] leading to presynaptic disturbance of the serotonergic system, or be secondary to the decrease in serotonergic afferents.

Several lines of evidence indicate that serotonergic dysfunction in AD has important functional consequences. In a study using retrospective data, loss in 5-HT_2A _binding has been confined to AD patients with aggressive symptoms [57]. When cognitive function is assessed *antemortem*, 5-HT_2A _receptors are lost in the frontal and temporal cortex only in patients with severe, but not with mild to moderate dementia [47]. The decrease in the expression of 5-HT_6 _receptors in the prefrontal cortex of AD patients can be correlated to the extent of aggressive behavior [5]. The number of 5-HT uptake sites is significantly reduced in the frontal and temporal cortex of AD patients with persistent depression, anxiety and overactivity, compared with AD patients without these symptoms [52], and 5-HT levels in the prefrontal cortex (Brodmann 10) of AD correlate with overactivity [2].

## Conclusion

Our results of normal aging patients, showing that 5-HT_2A _and 5-HT_6 _receptors are expressed by both pyramidal cells and stellate-shaped neurons, indicate that serotonergic fibers exert their influence upon these two neocortical cell types not only through the 5-HT_2A_, but also the 5-HT_6 _receptor. The significant 33–40% reduction in cells immunoreactive for 5-HT_2A _and 5-HT_6 _receptors observed in AD patients, affecting both large pyramidal cells and interneurons, points at a severely compromised serotonergic system in AD, involving not only serotonergic projection fibers, but also their corresponding receptors. This decline in receptors most likely contributes to the development of neuropsychiatric symptoms in AD.

## Authors' contributions

**Dietrich E. Lorke **performed the evaluation of the slides, interpreted the results and wrote the manuscript.**Gang Lu **performed immunohistochemistry and documented the results.**Eric Cho **performed the quantitative and statistical analyses. **David T. Yew **conceived and planned the study, performed the neuropathological examination and evaluated the slides.

## Abbreviations

AD: Alzheimer disease

BPSD: behavioral and psychological symptoms of dementia

5-HT: serotonin

5-HT_2A_: serotonergic receptor 2A

5-HT_6_: serotonergic receptor 6

NFT: neurofibrillary tangles

PBS: phosphate-buffered saline

SEM: standard error of the mean

## Figures and Tables

**Table 1:** Profiles of Alzheimer (AD) and matched normal aging patients

**Subject**	**Diagnosis**	**Sex**	**Age**	**Cause of death**
1	Normal	F	83	Pneumonia
2	Normal	F	86	Gastrointestinal bleeding
3	Normal	M	82	Pneumonia
4	Normal	M	93	Sudden death
5	Normal	F	72	Sudden death
6	Normal	F	85	Pneumonia
7	Normal	F	81	Pneumonia
8	Alzheimer	M	82	Subarachnoid hemorrhage
9	Alzheimer	F	91	Septicemia
10	Alzheimer	F	96	Pneumonia
11	Alzheimer	F	82	Pneumonia
12	Alzheimer	F	86	Pneumonia
13	Alzheimer	F	89	Sudden death
